# Noninvasive assessment of Ki-67 labeling index in glioma patients based on multi-parameters derived from advanced MR imaging

**DOI:** 10.3389/fonc.2024.1362990

**Published:** 2024-05-17

**Authors:** Ying Hu, Kai Zhang

**Affiliations:** ^1^ Department of Radiology, West China Hospital, Sichuan University, Chengdu, China; ^2^ West China Biomedical Big Data Center, West China Hospital, Sichuan University, Chengdu, China

**Keywords:** magnetic resonance imaging, diffusion tensor imaging, diffusion weighted imaging, perfusion imaging, magnetic resonance spectroscopy, glioma, Ki-67 labeling index

## Abstract

**Purpose:**

To investigate the predictive value of multi-parameters derived from advanced MR imaging for Ki-67 labeling index (LI) in glioma patients.

**Materials and Methods:**

One hundred and nine patients with histologically confirmed gliomas were evaluated retrospectively. These patients underwent advanced MR imaging, including dynamic susceptibility-weighted contrast enhanced MR imaging (DSC), MR spectroscopy imaging (MRS), diffusion-weighted imaging (DWI) and diffusion-tensor imaging (DTI), before treatment. Twenty-one parameters were extracted, including the maximum, minimum and mean values of relative cerebral blood flow (rCBF), relative cerebral blood volume (rCBV), relative mean transit time (rMTT), relative apparent diffusion coefficient (rADC), relative fractional anisotropy (rFA) and relative mean diffusivity (rMD) respectively, and ration of choline (Cho)/creatine (Cr), Cho/N-acetylaspartate (NAA) and NAA/Cr. Stepwise multivariate regression was performed to build multivariate models to predict Ki-67 LI. Pearson correlation analysis was used to investigate the correlation between imaging parameters and the grade of glioma. One-way analysis of variance (ANOVA) was used to explore the differences of the imaging parameters among the gliomas of grade II, III, and IV.

**Results:**

The multivariate regression showed that the model of five parameters, including rCBV_max_ (RC=0.282), rCBF_max_ (RC=0.151), rADC_min_ (RC= -0.14), rFA_max_ (RC=0.325) and Cho/Cr ratio (RC=0.157) predicted the Ki-67 LI with a root mean square (RMS) error of 0. 0679 (R^2^ = 0.8025).The regression check of this model showed that there were no multicollinearity problem (variance inflation factor: rCBV_max_, 3.22; rCBF_max_, 3.14; rADC_min_, 1.96; rFA_max_, 2.51; Cho/Cr ratio, 1.64), and the functional form of this model was appropriate (F test: p=0.682). The results of Pearson correlation analysis showed that the rCBV_max_, rCBF_max_, rFA_max_, the ratio of Cho/Cr and Cho/NAA were positively correlated with Ki-67 LI and the grade of glioma, while the rADC_min_ and rMD_min_ were negatively correlated with Ki-67 LI and the grade of glioma.

**Conclusion:**

Combining multiple parameters derived from DSC, DTI, DWI and MRS can precisely predict the Ki-67 LI in glioma patients.

## Introduction

Glioma is the most common neuroepithelial tumor of the cerebral nervous system (1). Ki-67 labeling index (LI) is a nuclear antigen expressed only by proliferating cells (2). Previous studies showed that Ki-67 LI was one of the vital biological behavior biomarkers in glioma and correlated with glioma grading and prognosis (3, 4).Therefore, accurate measurement of the Ki-67 LI is important for grading and synthesizing prognosis information in glioma.

Advanced MR imaging, such as dynamic susceptibility-weighted contrast enhanced imaging (DSC), diffusion-weighted imaging (DWI), diffusion tensor imaging (DTI) and magnetic resonance spectroscopic imaging (MRS), provide important information for evaluating tumors preoperatively. DSC magnetic resonance (MR) imaging is the most commonly used MR perfusion technique in clinical practice and is well established for evaluating relative cerebral blood volume (rCBV) and relative cerebral blood flow (rCBF) in brain tumors (5). Many studies have shown that the rCBV and the rCBF correlate with tumor grade and tumor vascularity (6, 7). Diffusion tensor imaging (DTI) can provide two quantitative parameters, namely mean diffusivity (MD) which is inversely correlated with tumor cellularity and grading in glioma (8) and fractional anisotropy (FA) (9). Recent studies demonstrated that the FA derived from DTI may correlate with tumor cellularity (10). Diffusion-weighted imaging (DWI) can noninvasively provide insight into the microscopic properties of tissues through evaluating Brownian movement of water, and the apparent diffusion coefficient (ADC) value can quantitatively reflect cellularity of the lesions (11, 12). MRS is a noninvasive tool which estimates the concentration of metabolites (13). Previous studies showed that choline (Cho)-containing compounds in tumors were considered to be markers for cell proliferation (14). Shimizu H and colleagues found a direct correlation between Ki-67 LI and Cho, Cho/Cr and Cho/NAA ratio (14).

To date, most of studies explored the correlation between individual parameters and Ki-67 LI. Few studies have combined multiple parameters to predict Ki-67 LI. Although there have been some efforts to combine advanced MR imaging in characterizing gliomas (15, 16). However, most of these studies focused on the grading of gliomas (17, 18) and few focused on cell proliferation or Ki-67 LI.

Therefore, the aim of this work was to investigate whether the multi-parameters derived from DSC, MRS, DWI and DTI technique can predict Ki-67 LI in glioma patients using stepwise multivariate regression.

## Materials and methods

### Participants

The institutional review board approved this retrospective study and waived the informed consent requirement. We retrospectively reviewed our institution’s database and identified 710 patients who underwent MR imaging for brain tumor evaluation from September 2018 to December 2023. Among these patients, 109 patients were finally enrolled for analysis according to the following inclusion criteria: a) patients were confirmed to have gliomas by pathologic analysis (excluded 357 subjects); b) The samples of pathologic analysis were from surgical resection (excluded 29 subjects); c) the reports of pathologic analysis included Ki-67 LI (excluded 39 subjects); d) The MR imaging were performed before any treatment (excluded 156 subjects); e) Their MR imaging had adequate image acquisition and without motion or susceptibility artifact (excluded 20 subjects). Therefore, 109 patients (61 men and 48 women, aged 4–80 years; mean age, 41.63 years) were finally evaluated.

### MR acquisition

MR acquisition were performed with a 3-T MR imaging system (Magnetom Skyra, Siemens Healthineers) with a twenty channel head and neck combined coil. All patients underwent conventional MR imaging and DTI, DWI, MRS and DSC imaging. The precontrast DTI protocol included TR/TE, 6000/93 ms; FOV, 230mm × 230mm; Matrix, 128 × 128; section thickness, 3 mm; voxel size, 1.8×1.8×3mm; number of section, 44; diffusion gradient encoding in 30 directions; b value, 1000 s/mm2.DWI scan parameters were as follows: TR/TE = 8200/102 ms; FOV, 230mm × 230mm; Matrix, 128 × 128; section thickness, 5 mm; b values = 0 and 1000 s/mm^2^. Multivoxel 2D MR spectroscopy was performed before the administration of contrast agent. The detailed imaging parameters for the MRS study were as follows: TR/TE, 1700/135 ms; flip angle 90°; section thickness, 10mm; FOV, 160mm×160mm; voxel size,10×10×10mm; coding phase, 16 ×16; Averages, 1. DSC MR perfusion imaging was performed by using a gradient-echo echo-planar sequence during the administration of 0.2 mmol/kg of gadoterate meglumine delivered with a power injector at a rate of 2ml/s followed by a 20ml bolus of saline administered at the same rate. Scan parameters were as follows: TR/TE, 1640 ms/30 ms; flip angle 90°; Averages, 1; FOV, 220 mm×220 mm; matrix 128 ×128; section thickness, 5mm; voxel size, 1.7×1.7×5mm; number of section, 21.

### Image processing and analysis

**Figure 1:**
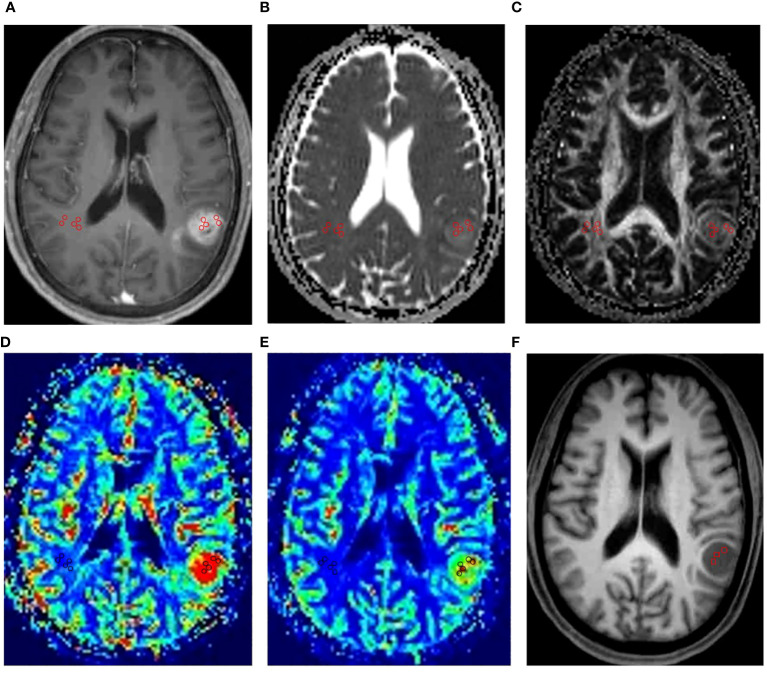
An example of ROI placement. This figure showed the ROI placement for a 62-year-old male patient with IDH1 wild-type grade IV glioma in the left temporal lobe. Firstly, we placed five circular regions of interest (ROIs) of 25mm^2^ to 30mm^2^ within the regions with the highest signal strength in the contrast-enhanced T1-weighted images **(A)**. Then, we copied the ROIs to the ADC maps **(B)**, FA maps **(C)**, CBV maps **(D)**, CBF maps **(E)**. Finally, five circular ROIs of same size from the above maps were placed in contralateral normal-appearing white matter. In MRS, the VOIs were placed in the structural MR imaging **(F)** within the solid portion of the tumor to avoid contamination from normal tissue or areas of necrosis, cysts or hemorrhage.

All imaging data were transferred from the scanner to a MMWP workstation (Siemens Healthcare, Erlangen, Germany) for postprocessing. For quantitative analysis, CBV maps, CBF maps, MTT maps, ADC maps, FA maps and MD maps were independently evaluated by two experienced neuro-radiologists who were blinded to the clinical and pathological information and any disagreements were resolved by consensus. The multi-parameters were calculated according to the method described in the previous studies (8, 19). The specific steps were as follows: a) Five circular regions of interest (ROIs) of 25mm^2^ to 30mm^2^ were carefully placed within the regions with the highest signal strength in the contrast-enhanced T1-weighted images to ensure the ROIs were placed in the solid component of a tumor and the normal tissue, the cystic, large necrotic, or hemorrhagic components of the tumor were avoided. These locations were then copied to the CBV maps, CBF maps, MTT maps, ADC maps, FA maps and MD maps; b) Five circular ROIs of same size from a) were placed in contralateral normal-appearing white matter. The mean value of these five ROIs was calculated as reference value; c) The highest, lowest, and mean CBV, CBF, MTT, ADC, FA and MD among the five ROIs acquired from a) were divided by the reference value to compute rCBV_max_, rCBV_min_, rCBV_mean_, rCBF_max_, rCBF_min_, rCBF_mean_, rMTT_max_, rMTT_min_, rMTT_mean_, rADC_max_, rADC_min_, rADC_mean_, rFA_max_, rFA_min_, rFA_mean_, rMD_max_, rMD_min_, rMD_mean_. An example of ROI placement was shown in **Figure 1**.

The spectra were automatically analyzed for the relative signal intensity (area under the fitted peaks in the time domain) of the following metabolites: Cho, Cr, NAA. The metabolite peaks were assigned at the following frequencies: choline (Cho) at 3.22 ppm, creatine (Cr) at 3.02 ppm, N-acetylaspartate (NAA) at 2.02 ppm. We selected one to three Volumes of interest (VOIs) (250mm^3^ to 300mm^3^) within the solid portion of the tumor to avoid contamination from normal tissue or areas of necrosis, cysts or hemorrhage based on conventional MR imaging as much as possible. The measured metabolites in these VOIs were averaged to represent the tumor. The ratios of Cho/Cr, Cho/NAA and NAA/Cr were finally calculated.

### Pathology

The histopathologic diagnosis was performed by pathologists and based on the WHO 2016 classification (20). The specimens were obtained from continuous sections after surgical resection. Surgical specimens were fixed in formalin and embedded in paraffin. The hematoxylin and eosin-stained specimens were checked to make the primary histopathological tissue diagnoses. The Ki-67 LI was obtained using the technique described in previous study (10, 21). Briefly, Ki-67 immunohistochemical staining was performed on paraffin embedded sections using the MIB-1 anti-human Ki-67 LI mouse monoclonal antibody (Dako, Carpinteria CA) at dilution of 1/600 and the EnvisionTM FLEX Targeted Retrieval System at high pH (Dako). Diaminobenzidine (DAB) was used as the chromogen. The Ki-67 LI was determined by calculating the percentage of MIB-1–positive tumor cell nuclei in a microscopic field containing approximately 400 to 500 tumor cells.

In each case, areas with the highest number of positive-staining tumor nuclei were selected for calculating the Ki-67 LI.

### Statistical analyses

Interobserver and intraobserver reliability coefficient of MRI parameters was assessed using intraclass correlation coefficients (ICC) with 95% confidence intervals (SPSS, version 20.0, IBM). All other statistical analyses were performed using stata (version,15.0). Firstly, the Pearson correlation was used to analyze the correlation between each parameter and Ki-67 LI respectively. Through correlation analysis, we screened out the imaging indicators that had the greatest correlation with Ki-67 LI among the maximum, minimum and mean values of CBF, CBV, MTT, ADC, FA and MD respectively. Using these indicators with high correlation with Ki-67 LI to represent tumors can reduce the possible mismatch between the location of pathological sampling and the placement of ROI or VOI. Therefore, these screened indicators and those obtained in MRS were used for subsequent statistical analysis.

Jones (22) pointed out that it is most appropriate to use multivariate linear regression to explore the predictive relationship between multiple parameters. In this study, Ki-67 LI was dependent variable, the image indicators were independent variables, age and sex were control variables.

The following was the mathematical formula and statistical process of the regression model of this study:


$$\mathrm{Regression equation}:{\text{Y}}_{\text{ik}}=\text{α}+{\text{β}}_{\text{ik}}{\text{X}}_{\text{ik}}+{\text{ε}}_{\text{ik}}\left(\text{i}=1\ldots \ldots 79;\text{k}=1\ldots \ldots \text{n}\right)$$

Where i is the sample size and k is the number of model’s independent variables. Yik is the predicted value of the dependent variable (Ki-67 LI) and Xik is the column vectors, which represents the independent variables. βik is the regression coefficient of the kth variable (ie, the prediction effect), and εik is the regression residual term, α is the intercept term of the regression equation. The above selected image indicators were gradually added into the model as independent variables according to the order of correlation with Ki-67 LI to form the predictive model of Ki-67 LI. We used the R2, RMSE, AIC and BIC to assess model quality.

We used the Ki-67 LI prediction model constructed above in the validation sample set to estimate the Ki-67 LI index for these subjects, and t test was used to compare whether there were differences between these predicted Ki-67 LI and the actual Ki-67 LI.

In addition, we analyzed the correlation between imaging indicators and the grade of glioma using the Pearson correlation analysis. We compared the differences of imaging indicators among the gliomas of grade II, III, and IV using one-way analysis of variance (ANOVA). *Post-hoc* tests using Bonferroni correction for multiple comparisons. Since there were only two subjects with glioma of grade I in this study, gliomas with tumor grade I were not included in the group comparison.

## Results

Among the 109 subjects included in this study, 79 subjects (age, 40.63 ± 16.82 years; age range, 4–80 years; female, 49; male, 30) were used as a dataset to construct the predictive model of Ki 67 LI, and 30 subjects (age, 43.16 ± 15.71 years; age range, 9–76 years; female, 12; male, 18) were used as a validation set for the predictive model. The information for the samples used to construct the Ki-67 LI prediction model was shown in **Table 1**. The average size of the ROIs which were placed within the solid component of the tumor were 26.3 ± 11.9mm^2^ for the neuroradiologist A and 28.75 ± 15.10 mm^2^ for the neuroradiologist B, respectively. There was no difference in the size of ROIs by the two neuroradiologists. The detailed size of ROIs placed by two neuroradiologists in each MRI maps were listed in **Table 2**. Intra-observer and inter-observer agreements for MRI parameters were good to excellent with ICCs ranging from 0.836 to 0.964 (**Table 3**).

**Table 1:** Patient demographic data characteristics.

Grade/Histology	IDH (Mut/WT)	Sex (Male/Female)	Age (Mean ± SD)	Ki-67 (Mean ± SD)
**Grade I (n=2)**	0/2	1/1	14 ± 9.899	0.045 ± 0.007
Pilocytic astrocytoma (n=2)	0/2	1/1	14 ± 9.899	0.045 ± 0.007
**Grade II (n=30)**	18/12	18/12	38.567 ± 16.425	0.071 ± 0.053
Diffuse astrocytoma (n=17)	7/10	11/6	35.688 ± 17.296	0.059 ± 0.040
Oligodendro-glioma (n=11)	11/0	7/4	41.09 ± 13.042	0.09 ± 0.068
Pleomorphic xanthoastrocytoma (n=2)	0/2	0/2	37 ± 31.113	0.055 ± 0.064
**Grade III (n=15)**	6/9	7/8	46.4 ± 9.326	0.189 ± 0.085
Anaplastic astrocytoma (n=8)	1/7	4/4	47.5 ± 10.085	0.169 ± 0.059
Anaplastic oligodendro-glioma (n=7)	5/2	3/4	45.143 ± 8.989	0.211 ± 0.108
**Grade IV (n=32)**	2/30	23/9	41.531 ± 18.719	0.301 ± 0.146
Glioblastoma (n=25)	1/24	18/7	46.8 ± 15.885	0.33 ± 0.141
Diffuse midline glioma (n=7)	1/6	5/2	21.875 ± 15.459	0.183 ± 0.12
**Sum (n=79)**	26/53	49/30	40.633 ± 16.822	0.186 ± 0.148

SD, standard deviation; IDH, Isocitrate dehydrogenase; Mut, IDH-mutant; WT, IDH-wild-type.

**Table 2:** The size of the ROIs or VOIs.

Parameter	Mean ± SD*	Mean ± SD*	P value
	Radiologists A	Radiologists B	
rCBV_min_	26.164 ± 2.012 (mm^2^)	27.149 ± 2.378 (mm^2^)	0.056
rCBV_mean_	27.235 ± 1.954 (mm^2^)	26.150 ± 2.320 (mm^2^)	0.061
rCBV_max_	26.567 ± 2.177 (mm^2^)	25.852 ± 2.543 (mm^2^)	0.053
rCBF_min_	27.419 ± 2.124 (mm^2^)	26.234 ± 2.491 (mm^2^)	0.066
rCBF_mean_	26.320 ± 2.066 (mm^2^)	26.535 ± 2.433 (mm^2^)	0.134
rCBF_max_	26.512 ± 2.289 (mm^2^)	27.112 ± 2.656 (mm^2^)	0.078
rMTT_min_	26.282 ± 2.138 (mm^2^)	26.876 ± 2.505 (mm^2^)	0.271
rMTT_mean_	26.183 ± 2.080 (mm^2^)	26.497 ± 2.447 (mm^2^)	0.186
rMTT_max_	27.315 ± 2.303 (mm^2^)	26.329 ± 2.570 (mm^2^)	0.053
rADC_min_	26.667 ± 2.251 (mm^2^)	26.818 ± 2.617 (mm^2^)	0.357
rADC_mean_	26.568 ± 2.193 (mm^2^)	27.213 ± 2.559 (mm^2^)	0.089
rADC_max_	26.137 ± 2.416 (mm^2^)	26.715 ± 1.782 (mm^2^)	0.092
rFA_min_	26.289 ± 1.993 (mm^2^)	26.165 ± 2.359 (mm^2^)	0.371
rFA_mean_	27.151 ± 1.935 (mm^2^)	26.166 ± 2.301 (mm^2^)	0.061
rFA_max_	26.583 ± 2.158 (mm^2^)	26.198 ± 2.524 (mm^2^)	0.376
rMD_min_	27.354 ± 2.105 (mm^2^)	26.550 ± 2.471 (mm^2^)	0.082
rMD_mean_	26.536 ± 2.047 (mm^2^)	26.551 ± 2.413 (mm^2^)	0.467
rMD_max_	27.568 ± 2.270 (mm^2^)	26.583 ± 2.636 (mm^2^)	0.079
Cho/Cr	272.18 ± 20.99 (mm^3^)	268.44 ± 2.014 (mm^3^)	0.183
Cho/NAA	264.01 ± 22.41 (mm^3^)	270.45 ± 25.83 (mm^3^)	0.299
NAA/Cr	274.33 ± 23.64 (mm^3^)	270.77 ± 28.06 (mm^3^)	0.357

*The values listed in this column were the size of the ROIs. ^#^The values listed in this column were the p-values of the T-test between the two neuro radiologists. The subscript “min” indicated the minimum value. The subscript “mean” indicated the mean value. The subscript “max” indicated the maximum value. rCBV, relative cerebral blood volume; rCBF, relative cerebral blood flow; rMTT, relative mean transit time; rADC, relative apparent diffusion coefficient; rFA, relative fractional anisotropy; rMD, relative mean diffusivity; Cho/Cr, choline/creatine; Cho/NAA, choline/N-acetylaspartate; NAA/Cr, N-acetylaspartate/creatine; SD, standard deviation.

**Table 3:** The results of correlation analysis and intra-class correlation coefficients.

Parameter	Mean ± SD*	r (P)#	ICC (95%CI)##	
			Inter-observer	Intra-observer
rCBV_min_	3.879 ± 3.054	0.357 (0.001)	0.931 (0.905–0.951)	0.964 (0.943–0.972)
rCBV_mean_	4.703 ± 2.686	0.755(<0.001)	0.925 (0.914–0.963)	0.958 (0.933–0.975)
rCBV_max_	6.023 ± 3.877	0.815 (<0.001)	0.942 (0.900–0.978)	0.953 (0.929–0.968)
rCBF_min_	3.495 ± 2.568	0.502 (<0.001)	0.946 (0.931–0.959)	0.949 (0.931–0.960)
rCBF_mean_	4.700 ± 2.641	0.755(<0.001)	0.920 (0.892–0.928)	0.923 (0.898–0.940)
rCBF_max_	5.866 ± 3.290	0.782 (<0.001)	0.909 (0.882–0.919)	0.918 (0.894–0.933)
rMTT_min_	1.044 ± 0.187	-0.184 (0.105)	0.897 (0.687–0.921)	0.911 (0.882–0.936)
rMTT_mean_	1.104 ± 0.627	-0.053 (0.64)	0.857 (0.633–0.914)	0.905 (0.892–0.943)
rMTT_max_	1.486 ± 0.200	-0.150 (0.188)	0.869 (0.662–0.932)	0.921 (0.878–0.956)
rADC_min_	1.400 ± 0.460	-0.657 (<0.001)	0.961 (0.943–0.972)	0.962 (0.944–0.973)
rADC_mean_	1.496 ± 0.510	-0.367 (<0.001)	0.933 (0.904–0.941)	0.936 (0.911–0.953)
rADC_max_	1.851 ± 0.808	-0.422 (<0.001)	0.922 (0.894–0.932)	0.931 (0.907–0.946)
rFA_min_	0.3 ± 0.145	0.787 (<0.001)	0.876 (0.666–0.900)	0.890 (0.861–0.915)
rFA_mean_	0.35 ± 0.183	0.778 (<0.001)	0.836 (0.612–0.893)	0.884 (0.871–0.922)
rFA_max_	0.414 ± 0.219	0.8 (<0.001)	0.848 (0.641–0.911)	0.901 (0.857–0.935)
rMD_min_	1.826 ± 0.142	-0.682 (<0.001)	0.940 (0.922–0.951)	0.941 (0.923–0.952)
rMD_mean_	1.998 ± 0.087	-0.533 (<0.001)	0.912 (0.883–0.920)	0.915 (0.890–0.932)
rMD_max_	2.188 ± 0.088	-0.548 (<0.001)	0.901 (0.873–0.911)	0.910 (0.886–0.925)
Cho/Cr	2.784 ± 2.014	0.627 (<0.001)	0.884 (0.675–0.908)	0.898 (0.869–0.923)
Cho/NAA	2.030 ± 1.271	0.402 (<0.001)	0.844 (0.621–0.901)	0.892 (0.879–0.930)
NAA/Cr	1.560 ± 1.183	0.086 (0.454)	0.856 (0.650–0.919)	0.908 (0.865–0.943)

*The values listed in this column were the measurements of Multi-parameters derived from MR Imaging. ^#^The values listed in this column were the results of correlation analysis between each parameter and ki-67 respectively. Data in parentheses were P values. ^##^Data in parentheses are the 95% confidence interval. The subscript “min” indicated the minimum value. The subscript “mean” indicated the mean value. The subscript “max” indicated the maximum value. rCBV, relative cerebral blood volume; rCBF, relative cerebral blood flow; rMTT, relative mean transit time; rADC, relative apparent diffusion coefficient; rFA, relative fractional anisotropy; rMD, relative mean diffusivity; Cho/Cr, choline/creatine; Cho/NAA, choline/N-acetylaspartate; NAA/Cr, N-acetylaspartate/creatine; SD, standard deviation; ICC, intraclass correlation coefficient; CI, confidence interval.

The results of the correlation analysis between each imaging indicators and Ki-67 LI showed that the rCBV_max_ (r=0.815, p<0.001), rCBF_max_ (r=0.782, p<0.001), rADC_min_ (r= -0.657, p<0.001), rFA_max_ (r=0.8, p<0.001), rMD_min_ (r=-0.682, p<0.001) had relatively high correlation with Ki-67 LI (**Table 3**). Therefore, the above indicators and ratios of Cho/Cr and Cho/NAA were included in subsequent stepwise regression analysis and group comparison. The ratio of NAA/Cr was not correlated with Ki-67 LI, so it was excluded from stepwise regression analysis.

The regression coefficients listed in our study were all non-standardized coefficients unless otherwise stated. The results of the stepwise regression analyses were as follows (**Table 4**): The model 1 showed that the regression coefficient of rCBV_max_ was 0.03 (P<0.001). In this model, the regression coefficients of age and sex were not statistically significant. Therefore, we excluded age and sex in the subsequent model construction. When the rFA_max_ was added, the model had higher R^2^ and lower RMSE, AIC and BIC, which means model 2 had better explanatory power for Ki-67 LI. In addition, the regression coefficient of rCBV_max_ was 0.03 (P<0.01). Thus, the model 1 overestimated the regression coefficient of rCBV_max._ We found that the model 3 had higher R^2^ and lower RMSE, AIC and BIC compared to model 2. The model 4 had higher increased R^2^ and lower RMSE, AIC and BIC compared to model 3. Therefore, model 3 was better than model 2, and model 4 was better than model 3. In model 4, the regression coefficient of rMD_min_ was not statistically significant. Therefore, we excluded rMD_min_ in the subsequent model construction. The model 5 had higher increased R^2^ and lower RMSE, AIC and BIC compared to model 4. The model 6 had higher increased R^2^ and lower RMSE, AIC and BIC compared to model 5. Therefore, model 5 was better than model 4, and model 6 was better than model 5. In addition, the value of regression coefficient of rCBV_max_, rFA_max_, rCBF_max_ were gradually decreased from model 1 to model 6 (**Table 4**), which means that the regression coefficients were overestimated in model 1 to model 5. The R^2^ in model 7 were similar with model 6. However, the RMSE, AIC and BIC were increased in model 7 compared to model 6 (**Table 4**). That is to say, the explanatory power of model 7 did not increase, but the simplicity of the model was affected compared to model 6.

**Table 4:** The results of stepwise multivariable regression.

Variables	Model 1	Model 2	Model 3	Model 4	Model 5	Model 6	Model 7
rCBV_max_	0.0300***	0.0189***	0.0140**	0.0133**	0.0125**	0.0108**	0.0108**
rFA_max_		0.299***	0.247**	0.245**	0.224**	0.219**	0.220**
rCBF_max_			0.0106**	0.00812	0.00926*	0.00677**	0.00680
rMD_min_				-0.101			
rADC_min_					-0.0427**	-0.0443**	-0.0444**
Cho/Cr						0.0115*	0.0117*
Cho/NAA							-0.000748
Age	0.00108						
Sex	-0.0219						
Constant	-0.0253	-0.0518***	-0.0636***	0.1411	0.0225	0.0199	0.0208
R_adj_^2^	0.6815	0.7584	0.7781	0.782	0.7875	0.8025	0.8025
RMSE	0.08511	0.07364	0.07103	0.07089	0.06999	0.06793	0.06839
AIC	-161.1932	-185.0233	-189.7623	-189.1394	-191.16	-194.9535	-192.966
BIC	-151.7154	-177.9149	-180.2845	-177.2921	-179.3128	-180.7368	-176.3799

The data listed in the table were non-standardized coefficients. rCBVmax, maximum relative cerebral blood volume; rCBFmax, maximum relative cerebral blood flow; rADCmin, minimum relative apparent diffusion coefficient; rFAmax, maximum relative fractional anisotropy; rMDmin, minimum relative mean diffusivity; Cho/Cr, the ration of choline and creatine; Cho/NAA, the ration of choline and N-acetylaspartate; NAA/Cr, the ration of N-acetylaspartate and creatine; RMSE, root mean square error; AIC, Akaike information criterion; BIC, Bayesian information criterion. “-” indicated that the variables in the column were not included in the row correspondence model.

***p<0.01, **p<0.05, *p<0.1

We could conclude that model 6 (Ki67 = 0.0199 + 0.0108rCBV_max_ + 0.219rFA_max_ + 0.00677rCBF_max_ + 0.0115Cho/Cr - 0.0443rADC_min_) may be the best model among these seven models. The standardized regression coefficients of each imaging indicator in this model were as follows: rCBV_max_ (RC= 0.282), rFA_max_ (RC=0.325), rCBF_max_ (RC=0.151), rADC_min_ (RC= -0.14), Cho/Cr (RC=0.157).

**Figure 2:**
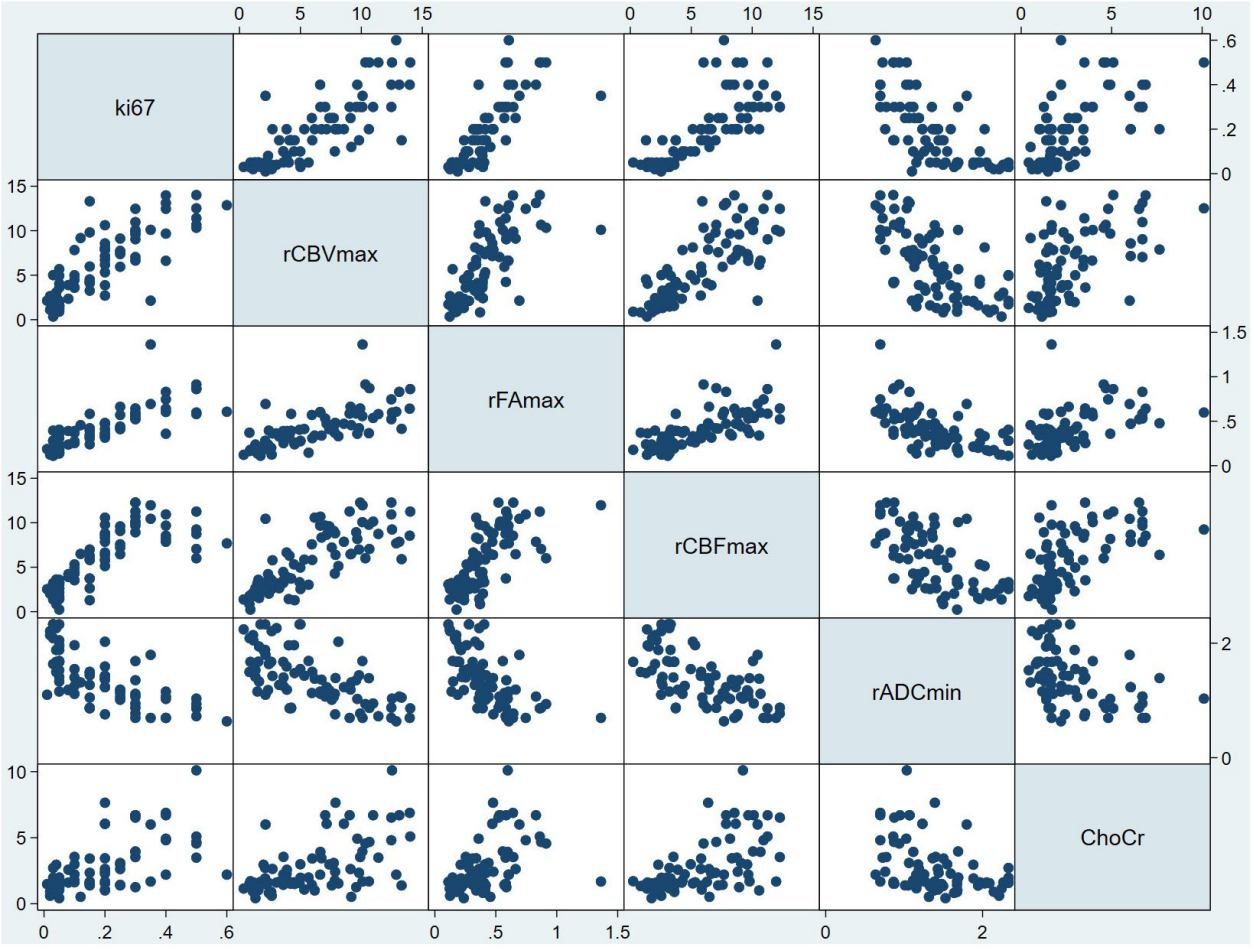
Scatterplot matrix of all variables in model 6. In each plot, the variable to the side of the graph was used as the Y variable, and the variable above and below the graph was used as the X variable. For example, in all the plots in the first column, the horizontal coordinate was Ki-67 LI, and the vertical coordinate from top to bottom was rCBVmax, the rFAmax, the rCBFmax, the rADCmin and Cho/Cr respectively. In addition, in all the plots in the first row, the vertical coordinate was Ki-67 LI, and the horizontal coordinate from left to right was rCBVmax, the rFAmax, the rCBFmax, the rADCmin and Cho/Cr respectively. From this figure, it can be seen that Ki-67 LI may have a positive correlation with rCBVmax, rFAmax, rCBFmax,Cho/Cr ration, while Ki-67 LI may have a negative correlation with rADCmin. ChoCr, Cho/Cr ration; rCBVmax, maximum relative cerebral blood volume; rCBF, relative cerebral blood flow; rADC_min_, minimum relative apparent diffusion coefficient; rFA_max_, maximum relative fractional anisotropy.

Then, we did regression check on model 6 and the results showed that there were no multicollinearity problem (Variance inflation factors of all independent variables were less than five: rCBVmax, 3.22; rCBFmax, 3.14; rADCmin, 1.96; rFAmax, 2.51; Cho/Cr ration, 1.64), which means that the model had no redundant information. In addition, the regression check demonstrated that the residuals were normally distributed (Shapiro-Wilk W normality test: z, 2.140; p, 0.016), which means that the model did not miss important variables. Finally, there was an appropriate functional form (Test for appropriate functional form: F, 0.502; p, 0.682). The scatterplot matrix showed that Ki-67 LI was positively linearly distributed with rCBVmax, rCBFmax, rFAmax and ratio of Cho/Cr respectively, while it was negatively linearly distributed with rADCmin (**Figure 2**). We used the Ki-67 LI prediction model obtained above to estimate the Ki-67 LI of the validation sample. There was no difference (P=0.087 for t test) between the estimation of Ki-67 LI (0.177 ± 0.126) estimate and the actual value of Ki-67 LI (0.186 ± 0.147) in the validation sample.

**Figure 3:**
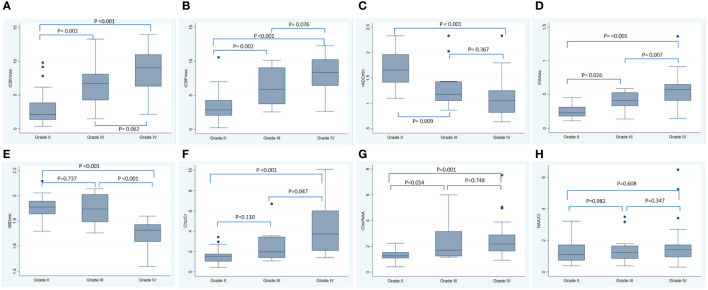
The box blots of various MRI metrics in different tumor grade. This figure showed the box plots for the rCBVmax **(A)**, the rCBFmax **(B)**, the rADCmin **(C)**, the rFAmax **(D)**, the rMDmin **(E)**, the Cho/Cr **(F)**, the Cho/NAA **(G)** and the NAA/Cr **(H)** in grade II, grade III and grade IV gliomas. The P-values listed in the picture were the results of *Post-hoc* tests using Bonferroni correction for multiple comparisons. rCBF, relative cerebral blood flow; rCBV, relative cerebral blood volume; rADC, relative apparent diffusion coefficient; rFA, relative fractional anisotropy; rMD, relative mean diffusivity; Cho/Cr, choline/creatine; Cho/NAA, choline/N-acetylaspartate; NAA/Cr, N-acetylaspartate/creatine; SD, standard deviation.

In addition, the analysis of the correlation between imaging indicators and the grade of glioma showed that the rCBVmax, rCBFmax, rFAmax, the ratio of Cho/Cr and Cho/NAA were positively correlated with the grade of glioma, while the rADCmin and rMDmin were negatively correlated with the grade of glioma (**Table 5**). The results of ANOVA showed that the rCBVmax, rCBFmax, rADCmin, rFAmax, rMDmin, the ration of Cho/Cr and Cho/NAA were different among grade II, III, and IV (**Table 5**). The *Post-hoc* tests showed that the rCBVmax, rCBFmax, rADCmin and rFAmax were different between grade II and grade III, the rCBVmax, rCBFmax, rADCmin, rFAmax, rMDmin, the ration of Cho/Cr and Cho/NAA were different between grade II and grade IV, and the rFAmax, rMDmin and the ration of Cho/Cr were different between grade III and grade IV (**Table 5**). The box blots of rCBVmax, the rCBFmax, the rADCmin, the rFAmax, the rMDmin, the ration of Cho/Cr, the Cho/NAA and the NAA/Cr in grade II, grade III and grade IV gliomas were showed in **Figure 3**.

**Table 5:** The results of correlation analysis between each parameter and grade and the results of group comparison.

Parameter	Mean ± SD			r (P)*	ANOVA#	Grade II vs Grade III #	Grade II vs Grade IV #	Grade III vs Grade IV #
	Grade II	Grade III	Grade IV					
rCBV_max_	3.055 ± 2.48	6.498 ± 3.193	8.738 ± 3.287	0.649 (<0.001)	<0.001	0.002	<0.001	0.062
rCBF_max_	3.416 ± 2.198	6.348 ± 2.700	8.159 ± 2.685	0.663 (<0.001)	<0.001	0.002	<0.001	0.076
rADC_min_	1.684 ± 0.366	1.303 ± 0.409	1.134 ± 0.377	-0.586 (<0.001)	<0.001	0.009	<0.001	0.367
rFA_max_	0.252 ± 0.095	0.401 ± 0.126	0.574 ± 0.23	0.630 (<0.001)	<0.001	0.026	<0.001	0.007
rMD_min_	1.913 ± 0.083	1.889 ± 0.113	1.702 ± 0.103	-0.704 (<0.001)	<0.001	0.737	<0.001	<0.001
Cho/Cr	1.549 ± 0.7	2.702 ± 1.832	4.055 ± 2.236	0.554 (<0.001)	<0.001	0.110	<0.001	0.047
Cho/NAA	1.323 ± 0.432	2.203 ± 1.406	2.473 ± 1.407	0.283 (0.012)	<0.001	0.054	0.001	0.748
NAA/Cr	1.32 ± 0.782	1.384 ± 0.903	1.715 ± 1.308	0.022 (0.849)	0.314	0.982	0.347	0.608

*The values listed in this column were the results of correlation analysis between each parameter and grade of glioma respectively. The P values were listed in parentheses. ^#^The values listed in these column were the P values of ANOVA and Post-hoc tests. rCBVmax, maximum relative cerebral blood volume; rCBFmax, maximum relative cerebral blood flow; rADCmin, minimum relative apparent diffusion coefficient; rFAmax, maximum relative fractional anisotropy; rMDmin, minimum relative mean diffusivity; Cho/Cr, the ration of choline and creatine; Cho/NAA, the ration of choline and N-acetylaspartate; NAA/Cr, the ration of N-acetylaspartate and creatine; SD, standard deviation.

## Discussion

This study estimated Ki-67 LI in glioma patients based on multi-parameters derived from DSC, DWI, DTI and MR spectroscopy imaging using multivariate regression and demonstrated that combining multiple parameters can precisely predict the Ki-67 LI. The model in our study with five dominant variables (rCBV_max_, rCBV_max,_ rADC_min_, rFA_max_ and Cho/Cr ratio) could predict Ki-67 LI with an R^2^ of 0. 8025 and a root mean square (RMS) error of 0.0679.

In addition, we found that rCBV_max_, rCBF_max_, rFA_max_, the ratio of Cho/Cr and Cho/NAA were positively correlated with Ki-67 LI and the grade of glioma, while the rADC_min_ and rMD_min_ were negatively correlated with Ki-67 LI and the grade of glioma.

The results about the correlation between various imaging indicator and Ki-67 LI and the grade of glioma in our study were generally agree with previous studies. Many studies reported a significant inverse correlation between ADC values or ADC ratio (lesion-to-normal) and Ki-67 LI (21, 22). Yan et al. (23) demonstrated that ADC was a reliable biomarker in predicting the proliferation level. This may be due to the level of ADC signal correlated with cell density in gliomas (23). MD measures the average motion of water molecules, independent of tissue directionality (24); it is considered a synonym of the coefficient of diffusion in different space guidelines (25). Therefore, our study also found that the rMD_min_ were negatively correlated with Ki-67 LI and the grade of glioma. Fractional anisotropy (FA) provides a quantitative estimation of diffusion anisotropy, and positive correlation was observed between the FA and Ki-67 LI in many studies (26, 27). George A. Alexiou and colleagues found significant negative correlation between the ADC ratio (lesion-to-normal ration) and the Ki-67 LI (rho = −0.545, p = 0.0087) and significant positive correlation between the FA ratio and the Ki-67 LI (rho = 0.489, p =0.02) (26). DSC imaging has been widely used to estimate CBV and CBF. Many studies reported a positive correlation between absolute or relative CBV and CBF values and cell density (28, 29). George A. Alexiou (26) and Anastasia K. Zikou (27) found strong correlation between rCBV and the Ki-67 LI in glioma (rho = 0.853, p < 0.0001) and in glioblastomas (r = 0.628, p = 0.07). Higher Cho metabolites at MR spectroscopy indicate increased membrane turnover and increased cellular density (30). However, Hiroaki Shimizu and colleagues showed that the Cho value tends to be underestimated in heterogeneous tumors resulting from intratumoral cyst, necrosis, hematoma, and indicate that the Cho value may no longer be reliable (14). Hiroaki Shimizu and colleagues demonstrated a linear relationship between the Ki-67 LI and Cho/Cr ratio (r =0.58, p = 0.02) and the Cho/NAA ratio (r= 0.60, p=0.02) (16). The regression coefficients between Ki-67 LI and rCBV_max_, rADC_min_, rFA_max_ and Cho/Cr ration in our study were relatively lower in our study compared with previous studies. The inconsistency may be due to the differences in statistical analyses. They performed univariate linear regression analysis which may lead to miscalculation of regression coefficients resulting from missing important variables. The above showed that diffusion, perfusion and spectroscopy imaging can be used to assess vascularity, metabolic activity, biochemical concentration and cellularity. These may be the reasons for the correlation between the parameters obtained in advanced MRI and the Ki-67 LI and the grade of glioma in this study.

We are not aware of previous work presenting Ki-67 predictive models based on multi-parameters derived from MR imaging using stepwise multivariate regression. Recently, Evan D. H. Gates and colleagues estimated Ki-67 maps using multi-parameters and reported the random forest algorithm best modeled Ki-67 with 4 imaging inputs (T2-weighted, fractional anisotropy, cerebral blood flow, Ktrans) and with a RMSE of 0.035 (R^2^ = 0.75) (4). In our study, the model with also 5 variables (rCBV_max_, rCBF_max_, rADC_min_, rFA_max_ and Cho/Cr ration) predicted Ki-67 LI with a RMSE of 0.0679 (R^2^ = 0.8025). The RMSE in our research was slightly larger, the reason maybe the MR sequences and statistical analyses were different between our and their study which may lead to some differences in results. However, our model was tested by regression diagnosis which showed there was an appropriate functional form and the model did not miss important variables. In addition, we also tested in the validation set that there was no statistical difference between the Ki-67 LI evaluated by the predictive model constructed in this study and the actual Ki-67 LI. Therefore, our model also had important clinical value in noninvasively predicting the Ki-67 LI.

Ki-67 LI, a tumor cell proliferation index, is a widely recognized biomarker for quantitative evaluation of glioma growth and prognosis of patients (31). The Ki-67 LI prediction model constructed in this study will lead to more accurate characterization of tumors and allows us to distinguish between high-proliferating and low-proliferating gliomas. Such features afford additional presurgical information to the conventional morphological images. In clinical application, we suggest that advanced magnetic resonance examination, especially DTI, DWI, DSC and MRS imaging, be performed before surgery in glioma patients, and combine the model in our study to predict Ki-67 LI before surgery to noninvasive evaluation of pathological features of glioma.

This study has several limitations. Firstly, this was a retrospective research and only DSC, DWI, DTI and MR spectroscopy imaging were analyzed. In the future, more advanced MR imaging techniques need to be included to verify the results of this study. Second, the relation between the ROIs placement on the parameter maps of MR imaging and the histologic sampling used for the proliferation analysis remains unclear, although Ki-67 LI was determined in the highest density of stained areas. Another limitation was the heterogeneity of Ki-67 LI in glioma. The Ki-67 LI of the same lesion in the same patient in different areas was very different, although we try to enroll the maximum of Ki-67 LI in the section in this study, and select the ROI representing the most serious lesions in the image, so as to achieve the match between MR image and pathology as much as possible. The third limitation is that all MRI scans were performed on a single machine, which can avoid errors due to different machines, but it is also impossible to know whether the models constructed in this study will be applicable on other MRI machines. In the future, it is necessary to include more patients scanned on different MRI machines to verify whether the model obtained in this study is applicable to other machines, or to build a standardized model that can be applied to different MRI machines. The fourth limitation is that due to the limited sample size, the prediction model of glioma histological type was not constructed in this study, and the prediction model of glioma Ki-67 LI was not constructed according to the histological types. It may be possible to get a more accurate predictive model by building models based on histological types. Therefore, a larger sample size including various histological types will be needed in the future to complete this work. Finally, there are many new methods for feature extraction of magnetic resonance data, such as texture analysis. It is unknown whether the magnetic resonance parameters obtained by these new methods can build a more reliable prediction model for Ki-67 LI. Although the magnetic resonance parameters obtained in this study are more convenient compared with texture analysis, it is of great value to use more new magnetic resonance parameters to construct the Ki-67 LI model, and compare the differences between the model obtained in this study and the model obtained by the new method, or standardize the model between the parameters obtained by the traditional method and the parameters obtained by the new method.

In conclusion, we found that rCBV_max_, rCBF_max_, rADC_min_, rFA_max_ and Cho/Cr ratio are correlated to Ki-67 LI in glioma patients. At the same time, combining multiple parameters derived from DSC, DWI, DTI and MRS can precisely predict the Ki-67 LI in glioma patients. This will allow us to noninvasively evaluate the pathological features and predict the prognosis of patients with glioma before surgery, and provide some information for the selection of clinical treatment.

## Data availability statement

The raw data supporting the conclusions of this article will be made available by the authors, without undue reservation.

## Ethics statement

The studies involving humans were approved by West China hospital of Sichuan University. The studies were conducted in accordance with the local legislation and institutional requirements. The participants provided their written informed consent to participate in this study.

## Author contributions

YH: Writing – review & editing, Writing – original draft, Visualization, Validation, Supervision, Software, Resources, Project administration, Methodology, Investigation, Funding acquisition, Formal analysis, Data curation, Conceptualization. KZ: Writing – review & editing, Visualization, Software, Resources, Project administration, Methodology, Investigation, Formal analysis, Data curation.
